# Systemic Inflammatory Response Index as a Predictor of Postoperative Infectious Complications in Elderly Patients Undergoing Posterior Spinal Instrumentation

**DOI:** 10.3390/jcm14217632

**Published:** 2025-10-28

**Authors:** Anil Agar, Sefa Key, Hamza Yavuz

**Affiliations:** Department of Orthopedics, Firat University Hospital, Firat University, Elazig 23119, Turkey; skey@firat.edu.tr (S.K.); drhamzayavuz23@gmail.com (H.Y.)

**Keywords:** systemic inflammatory response index (SIRI), osteoporotic vertebral fracture (OVF), lumbar spinal stenosis (LSS), posterior instrumentation, spine surgery, inflammation, elderly patients

## Abstract

**Objective:** To assess the predictive value of systemic inflammatory markers for postoperative complications in older adults undergoing posterior spinal instrumentation for either lumbar spinal stenosis (LSS) or osteoporotic vertebral fractures (OVFs). This study design as a retrospective observational study. **Methods:** Fifty-four patients aged ≥ 55 years who underwent posterior spinal instrumentation between 2020 and 2023 were retrospectively analyzed. Patients were grouped into LSS (n = 27) and OVF (n = 27) cohorts. Preoperative, early postoperative, and 6-month follow-up systemic inflammatory marker levels, including the Systemic Inflammatory Response Index (SIRI), Systemic Immune-Inflammation Index (SII), Neutrophil-to-Lymphocyte Ratio (NLR), Platelet-to-Lymphocyte Ratio (PLR), and Monocyte-to-Lymphocyte Ratio (MLR), were recorded. The primary outcome was the occurrence of postoperative infectious complications. ROC curve analysis was conducted to evaluate the discriminatory power of each marker. **Results:** SIRI values were significantly higher in the OVF group than in the LSS group at all time points (*p* < 0.05). Postoperative complications occurred in 7.4% of patients, equally distributed between groups. ROC analysis showed that preoperative SIRI had the highest predictive value (AUC = 0.743), with a cutoff value of 2.69 yielding 100% sensitivity and 65.3% specificity. Other indices showed poor predictive accuracy (AUC < 0.70). **Conclusions:** Preoperative SIRI is a promising, easily obtainable biomarker for identifying older patients at higher risk of postoperative complications following posterior spinal instrumentation. Its implementation may improve preoperative risk stratification in spine surgery.

## 1. Introduction

Degenerative lumbar spinal stenosis (LSS) and osteoporotic vertebral fractures (OVFs) are prevalent causes of morbidity and reduced quality of life among older adults [1,2]. With the global trend of population aging, surgical interventions for these conditions, particularly posterior spinal instrumentation, have markedly increased [3,4,5]. Despite advancements in surgical techniques and perioperative care, older patients undergoing spine surgery continue to face a high risk of postoperative complications due to the effects of comorbidities and age-related physiological decline [6,7,8,9].

Systemic inflammation plays a pivotal role in the pathogenesis of both degenerative spinal disorders and osteoporotic fractures, including OVFs [10,11]. Inflammatory responses reflect not only underlying disease activity but also tissue healing capacity and immune resilience following surgical trauma [12]. Different metrics such as operative time, preoperative length of stay, hospital stay, blood loss, incision length, and postoperative pain scores have been used to compare the invasiveness of various procedures [13,14]. Platelets and leukocytes are central to a defensive inflammatory response, and increased platelet and leukocyte counts have been widely associated with stress, trauma, ischemia, and infection [15,16,17]. Given their elevated levels in early fracture hematoma, neutrophils are known to contribute to fracture healing, initiating the downstream primary immune response and removing damage-associated molecular patterns (DAMPs) and microbe- or pathogen-associated molecular patterns (MAMPs) in nonsterile skin wounds [18]. Consequently, preoperative and postoperative inflammatory status has gained increasing attention as a prognostic factor in spine surgery.

Recently, composite hematologic markers based on routine blood counts have been developed as simple and cost-effective indicators of systemic inflammation. The systemic inflammatory response index (SIRI) [calculated as (neutrophils × monocytes/lymphocytes)] and the systemic immune-inflammation index (SII) [calculated as (platelets × neutrophils/lymphocytes)] can independently predict adverse outcomes in cardiovascular disease, cancers, and orthopedic surgery [19,20,21]. Conventional inflammatory ratios, including the neutrophil-to-lymphocyte ratio (NLR), platelet-to-lymphocyte ratio (PLR), and monocyte-to-lymphocyte ratio (MLR), are markers of systemic inflammatory and immunologic response and are associated with postoperative complications and mortality in several surgical groups [22,23,24,25].

Although many studies have evaluated their utility in predicting the outcomes of hip fracture surgery and tumor-related spine surgery, few have analyzed them in the context of spine surgery, particularly among older patients with OVF or LSS [26,27,28].

This study aimed to evaluate the aforementioned inflammatory marker profiles in older patients undergoing surgery for OVF or LSS. A secondary aim was to examine the relationship between these markers and postoperative complications, with the goal of identifying prognostic indicators that could help with perioperative risk stratification.

## 2. Materials and Methods

### 2.1. Study Design and Patient Selection

This retrospective observational study was conducted at a single tertiary care institution and was approved by the ethics committee of our institute (approval no. 34464, date: 14 May 2025). Between January 2020 and January 2023, 98 patients underwent posterior spinal instrumentation at our hospital. Patients were divided into OVF and LSS groups to explore potential differences in systemic inflammatory responses arising from distinct underlying pathologies—mechanical degeneration versus osteoporotic fragility fractures. All patients received a standardized perioperative regimen, including prophylactic intravenous cefazolin (2 g preoperatively and continued for 24 h postoperatively), multimodal analgesia with acetaminophen and NSAIDs (unless contraindicated), and no routine corticosteroid administration. We applied the following inclusion criteria: being aged ≥55 years; having undergone posterior spinal instrumentation for LSS or OVF; having laboratory data available at 3 time points (preoperative, i.e., within 7 days prior to surgery; early postoperative, i.e., within 72 h after surgery; and at the 6-month follow-up); having preoperative bone mineral density measurements via dual-energy X-ray absorptiometry; having ≥6 months of clinical and radiological follow-up; and having a complete assessment of postoperative complications throughout the follow-up period. Patients with spinal infections, malignancies, or vertebral trauma unrelated to osteoporosis, as well as those with a history of revision spine surgery, autoimmune or systemic inflammatory diseases, active infections at the time of surgery, or insufficient laboratory or follow-up data, were excluded.

All patients underwent posterior instrumentation of ≤3 vertebral segments, and all surgeries were performed by the same orthopedic spine team using a standardized posterior midline approach, ensuring procedural uniformity across cases.

### 2.2. Data Collection and Laboratory Parameters

Demographic data, operative indications, surgical levels, ASA (American Society of Anesthesiologists) scores and comorbidities were recorded. Laboratory values were obtained at the aforementioned time points: preoperative, early postoperative, and at the 6-month follow-up. Complete blood counts were used to calculate the Systemic Inflammatory Response Index (SIRI = neutrophils × monocytes/lymphocytes), Systemic Immune-Inflammation Index (SII = platelets × neutrophils/lymphocytes), Neutrophil-to-Lymphocyte Ratio (NLR), Platelet-to-Lymphocyte Ratio (PLR), and Monocyte-to-Lymphocyte Ratio (MLR).

All patient’s medical comorbidities were noted. These included dementia, cerebrovascular disease, diabetes mellitus, chronic obstructive pulmonary disease, coronary artery disease, chronic renal failure, congestive heart failure, hypertension, dementia, and malignancy. In order to calculate the Charlson Comorbidity Index, it was considered sufficient to refer to an existing disease in the records.

### 2.3. Assessment of Complications

All patients received clinical and radiological follow-up for ≥6 months. Postoperative complications were recorded and categorized as infectious complications (surgical site infection, urinary tract infection, and pneumonia), neurological complications (new or worsening deficits), mechanical complications (instrumentation failure and adjacent segment fracture), or systemic complications (deep vein thrombosis, pulmonary embolism, and cardiac events).

### 2.4. Statistical Analysis

SPSS v22 (IBM, Armonk, NY, USA) was used for all statistical analyses. Descriptive statistics are presented as means ± standard deviations for continuous variables and frequencies and percentages for categorical variables. The normality of data distribution was evaluated using the Shapiro–Wilk test. Between-group comparisons were performed using the independent-samples *t*-test for normally distributed variables and the Mann–Whitney U test for nonnormally distributed variables. The Fisher exact test was used to compare categorical variables, such as complication rates. Given the low event rate (4/54), we anticipated small-sample bias and separation in multivariable models. We therefore fitted a multivariable logistic regression with postoperative infectious complication (yes/no) as the dependent variable and preoperative SIRI, age, ASA class, CCI, and group (OVF vs. LSS) as covariates. Because the maximum-likelihood model showed signs of separation and unstable confidence intervals, we additionally applied an L2-penalized (ridge) logistic regression to obtain stabilized coefficient estimates. Results are reported as adjusted odds ratios (ORs) for the penalized model; conventional *p*-values and confidence intervals were not emphasized due to penalization and sparse events. Receiver operating characteristic (ROC) curve analyses were performed to assess the predictive performance of the inflammatory indices (SIRI, SII, NLR, PLR, and MLR) in identifying complications. To determine the ideal threshold for each marker, the area under the curve (AUC), sensitivity, specificity, and Youden index were computed. Statistical significance was defined as *p* < 0.05.

## 3. Results

**Figure 1:**
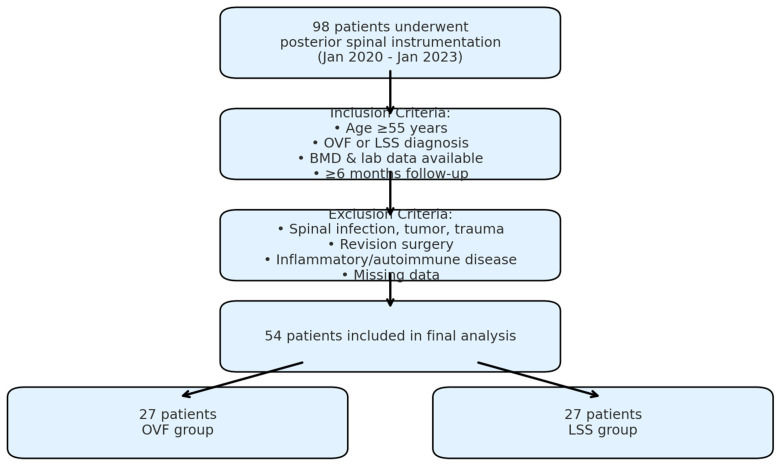
Flow diagram of patient selection and grouping.

After applying the inclusion and exclusion criteria, 54 patients were included in the analysis, 27 of whom underwent posterior spinal instrumentation for OVF (the OVF group) and 27 for LSS (the LSS group) (Figure 1). The baseline characteristics of the groups are given Table 1.

The patients’ mean age was 67.4 ± 5.9 years (range: 56–79 years). All patients had average follow-up period was 10.3 months (range: 6–17 months). Perioperative and postoperative complications were assessed for all patients. Preop, postop and follow-up inflammatory marker values of all patients are given in Table 2.

**Figure 2:**
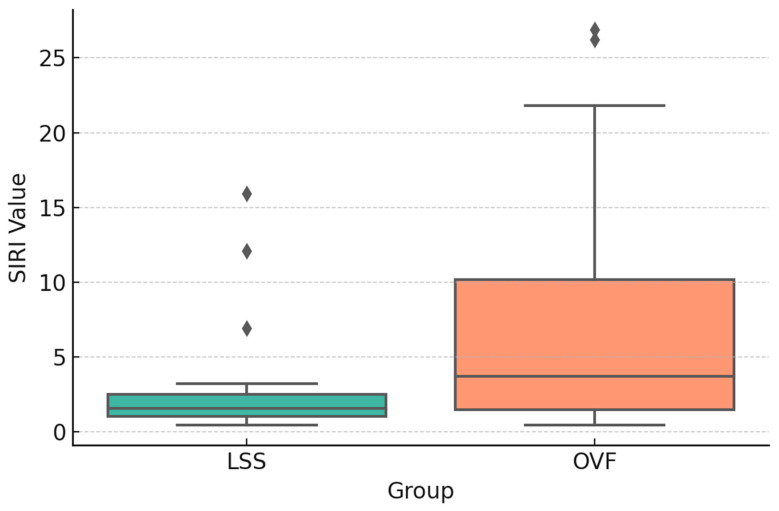
Boxplot comparison of preoperative SIRI values between groups (*p*: 0.0178).

**Figure 3:**
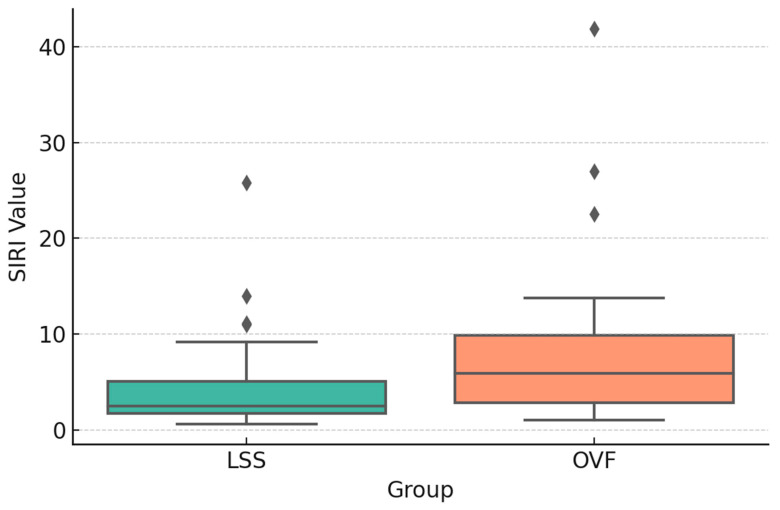
Boxplot comparison of early postoperative SIRI values between groups (*p*: 0.0147).

**Figure 4:**
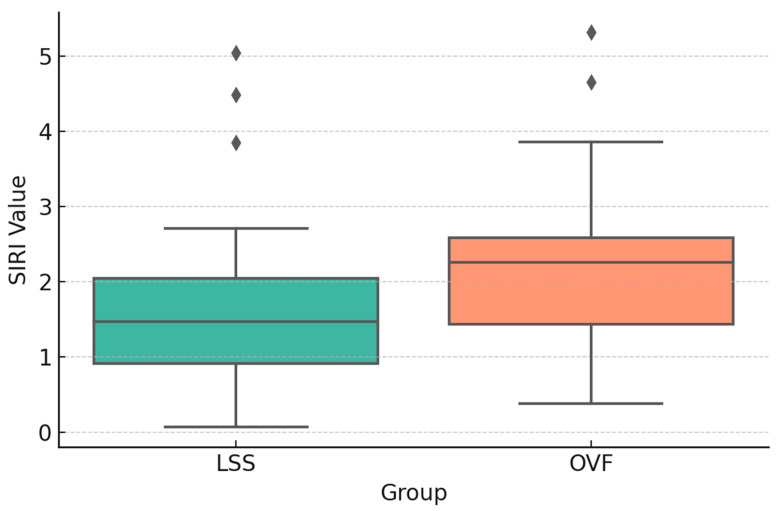
Boxplot comparison of 6-month follow-up SIRI values between groups (*p*: 0.0429).

SIRI values were significantly higher in the OVF group than in the LSS group preoperatively (1.92 ± 0.71 vs. 1.45 ± 0.55; *p* = 0.01) (Figure 2), postoperatively (2.21 ± 0.79 vs. 1.67 ± 0.66; *p* = 0.01) (Figure 3), and at the 6-month follow-up (1.63 ± 0.68 vs. 1.28 ± 0.50; *p* = 0.04) (Figure 4). No significant between-group differences were observed for the other indices (SII, NLR, PLR, or MLR; all *p* > 0.05).

Four patients (7.4%) experienced postoperative complications: surgical site infection occurred in 2 patients in each group. No significant difference in the complication rate was noted between the LSS and OVF groups.

Spearman’s correlation analysis revealed no significant association between either Charlson Comorbidity Index or ASA scores and the presence of postoperative complications. The correlation between CCI and complications was weak and negative (ρ = −0.147, *p* = 0.287), while the correlation between ASA score and complications was also weak and negative (ρ = −0.081, *p* = 0.560). These findings indicate that higher comorbidity burden or anesthetic risk grade was not significantly related to the occurrence of postoperative complications in this cohort.

**Figure 5:**
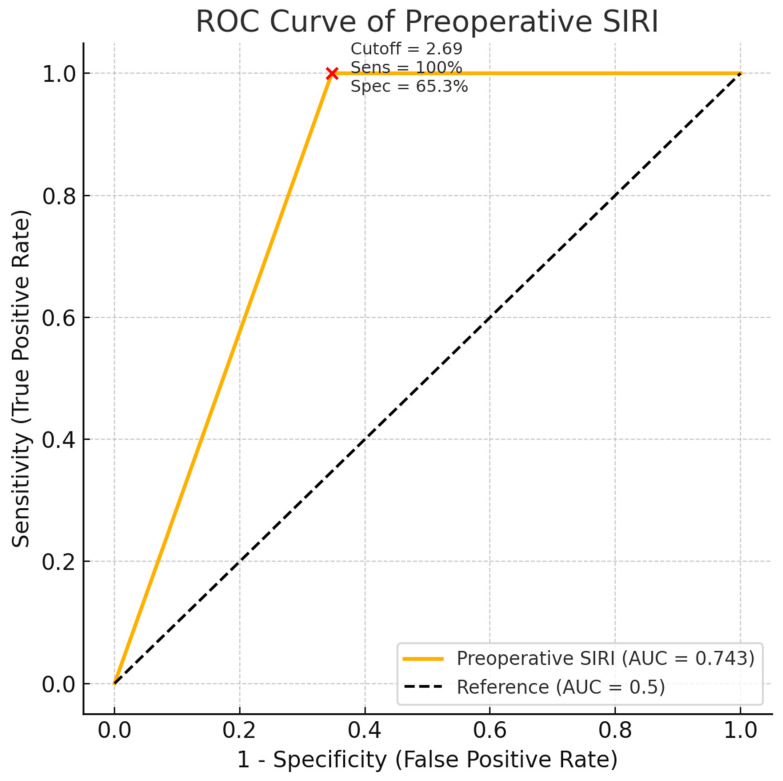
Receiver Operating Characteristic (ROC) curve of preoperative SIRI for predicting postoperative complications.

ROC analysis revealed that of all the indices, preoperative SIRI had the highest predictive value, with an AUC of 0.743 (95% CI 0.608–0.878) and an optimal cutoff of 2.69 (sensitivity: 100%, specificity: 65.3%, Youden index: 0.653). With AUC < 0.70, other parameters, such as preoperative SII, NLR, PLR, and MLR, demonstrated poor discriminatory ability (Figure 5).

In multivariable analysis including preoperative SIRI, age, ASA, CCI, and group, the maximum-likelihood model was unstable owing to quasi-separation. In the L2-penalized logistic regression, preoperative SIRI was not independently associated with postoperative infectious complications (adjusted OR ≈ 0.99). Age and group also showed ORs close to unity. ASA and CCI displayed inflated/shrunken coefficients consistent with separation, supporting the decision to use penalization.

## 4. Discussion

This study assessed systemic inflammatory indices in older patients with posterior spinal instrumentation for LSS or OVF and evaluated the association between these indices and postoperative complications. As the number of older patients requiring spine surgeries increases, identifying easy-to-use preoperative risk indicators is essential for improving postoperative outcomes.

Inflammatory systemic biomarkers have been applied in orthopedics to predict postoperative complications, such as acute deep vein thrombosis [29] and periprosthetic joint infections [30] as well as to evaluate the severity of various fracture types [31,32]. Our results revealed that SIRI values were significantly higher in the OVF group than in the LSS group at all 3 time points—preoperative, early postoperative, and at the 6-month follow-up. Although SIRI values were consistently higher in the OVF group, this did not correspond to a higher rate of postoperative infections. This suggests that the elevated SIRI levels in osteoporotic fracture patients may reflect a chronic low-grade inflammatory and frailty-related state, rather than an acute predisposition to infection following surgery. This is likely due to the chronic inflammatory state associated with osteoporotic fractures, which are not only mechanical events but are also linked to underlying frailty, immune dysfunction, and systemic inflammation.

Prior research has demonstrated the prognostic value of SIRI and SII in various diseases. A cutoff of 3.99 for SIRI and 596.91 for SII was reported by Zhao et al. [33] to predict problems following aortic dissection. The cutoffs for predicting pneumonia associated with stroke were 1384 for SII and 2.676 for SIRI [34]. The cutoffs for SIRI and SII in predicting prognosis following hepatocellular cancer were 1.25 and 179.8, respectively [35] and for predicting death after COVID-19 were 2.93 and 1835, respectively [36]. With a sensitivity of 88.2% and a specificity of 74.4%, the SIRI score cutoff value for predicting delirium following hip replacement was 0.987 [37]. Among the inflammatory markers evaluated, preoperative SIRI performed best in predicting postoperative complications with an AUC of 0.743 and a cutoff value of 2.69, indicating a fair discriminatory power. While the identified SIRI cutoff of 2.69 achieved high sensitivity (100%), its modest specificity (65.3%) suggests that false positives may occur if used alone for screening. Clinically, such a cutoff may be better suited for identifying patients who warrant closer postoperative surveillance rather than serving as a standalone exclusionary tool. Our findings are thus consistent with previous studies that have identified SIRI as a promising prognostic marker in various surgical and oncologic settings, as it combines the effects of neutrophilia and monocytosis with lymphopenia, both of which are hallmarks of systemic stress and poor immunological reserve [38,39].

In contrast, SII, NLR, PLR, and MLR did not show significant associations with complications in our cohort. Although these markers have demonstrated prognostic value in the orthopedic, oncologic, and cardiovascular literature [40,41], the low number of complications (n = 4) in our study may have limited the statistical power to detect subtle differences. Larger cohorts with higher event rates may thus be needed to validate their use in elective spine surgery.

Although the OVF group had higher inflammatory indices, no significant between-group difference in complication rates was noted. This may be attributed to the limited extent of surgery (≤3 levels of instrumentation) and consistent perioperative care. Nonetheless, the influence of unmeasured variables, such as nutritional status, frailty scores, sarcopenia, and medication use, cannot be excluded.

Notably, our findings also have economic significance. The inflammatory markers we studied are measured from routine blood counts conducted when the patient is hospitalized and thus do not incur additional costs or require specialized equipment or extended turnaround times (a few minutes vs. ≥24 h, depending on the type of cytokine), unlike proinflammatory cytokines.

This study has several limitations. First, its retrospective design limits causal inference and introduces potential selection bias. Second, the low event rate and small sample size precluded subgroup analyses or strong statistical modeling. Given the small sample size and the limited number of complication events (n = 4), the ROC analysis may be prone to overfitting. We acknowledge that the reported sensitivity may not generalize to larger populations. A post hoc power analysis using G*Power (version 3.1.9.7; Heinrich-Heine-University Düsseldorf, Germany) indicated that a minimum of 162 patients would be required to achieve 80% power at an alpha of 0.05. Therefore, larger multicenter studies are needed to validate these findings. Given the small number of events, the ROC findings should be interpreted cautiously, as the 100% sensitivity may reflect overfitting. Although SIRI demonstrated moderate discriminatory power (AUC = 0.743), this value requires validation in larger or bootstrapped cohorts. Multivariable modelling in this small cohort with very few events was underpowered and prone to separation, limiting the precision of effect estimates. After penalization, preoperative SIRI did not emerge as an independent predictor of infection when adjusting for age, ASA, CCI, and group, suggesting that the univariable discriminatory signal (AUC = 0.743) should be interpreted with caution. Future studies should employ larger samples with more outcome events and consider bootstrapping, Firth bias-reduction, or propensity score methods to validate the stability of SIRI thresholds and multivariable effects. Third, there was no information on potential confounders, such as body composition, history of inflammatory diseases, or intraoperative blood loss. Important potential confounders such as frailty indices, nutritional parameters, sarcopenia, and medication use (e.g., corticosteroids or immunosuppressants) were not assessed. Future research integrating these variables with inflammatory markers may yield more comprehensive risk models for elderly surgical patients. Fourth, the single-center design may hinder our results’ broader applicability. Because all patients were treated at a single institution by the same surgical team, our results may not fully represent broader surgical populations. Differences in perioperative protocols, patient demographics, and health systems could influence the external validity of our findings. Future larger, prospective, multicenter cohort studies are warranted to validate the preoperative risk stratification ability not only of inflammatory indices, such as SIRI, but also their combination with clinical and radiological characteristics in multivariable prediction models. Additionally, investigating strategies to lessen systemic inflammation, such as pharmacological modulation, nutritional support, and rehabilitation, may yield additional clinical advantages.

## 5. Conclusions

Our results revealed a significantly higher preoperative SIRI in older patients receiving posterior spinal instrumentation for OVF than for LSS. Additionally, higher preoperative SIRI indicated good predictive ability and was associated with a higher risk of postoperative complications. These findings suggest that SIRI has potential as an affordable and readily available biomarker for perioperative risk assessment in spine surgery. While preoperative SIRI showed fair univariable discrimination, it was not an independent predictor after multivariable adjustment in this small, low-event cohort. Given the limited number of events, the discriminatory performance of SIRI should be interpreted cautiously. These findings are exploratory and require validation in larger, prospective multicenter studies before clinical implementation. Future larger-scale prospective studies are required to validate these findings and support the incorporation of SIRI and inflammatory markers into standard surgical decision-making algorithms. In future research, integrating SIRI with validated frailty or nutritional risk indices (e.g., modified frailty index, CONUT score) could enhance preoperative risk stratification frameworks. Such composite models may improve prediction accuracy beyond conventional clinical scoring systems alone.

## Figures and Tables

**Table 1:** Baseline Characteristics of the LSS and OVF Groups.

Characteristic	LSS (n = 27)	OVF (n = 27)	Total (n = 54)	*p* Value
Age (mean ± SD)	66.4 ± 5.9	65.7 ± 4.2	66.1 ± 5.1	0.6
Female, n (%)	16 (59.3%)	18 (66.7%)	34 (63.0%)	0.7
Male, n (%)	11 (40.7%)	9 (33.3%)	20 (37.0%)	0.7
Complications, n (%)	2 (7.4%)	2 (7.4%)	4 (7.4%)	1
Follow-up (months, mean ± SD)	10.4 ± 4.1 (6–17)	10.1 ± 3.9 (7–16)	10.3 ± 3.5 (6–17)	0.7
Charlson comorbiditiy index (mean ± SD)	3.07 ± 0.66	3.04 ± 0.53	3.06 ± 0.60	0.8
ASA score (mean ± SD)	2.82 ± 0.39	2.88 ± 0.33	2.85 ± 0.36	0.7

Mann–Whitney U test, Independent samples *t*-test (*p* < 0.05).

**Table 2:** Comparison of Inflammatory Markers Between Fracture and Stenosis Groups.

Parameter	OVF (Mean ± SD)	LSS (Mean ± SD)	*p*-Value
Preop SIRI	7.03 ± 7.83	2.69 ± 3.54	0.0178 *
Postop SIRI	8.50 ± 9.13	4.69 ± 5.49	0.0147 *
Follow-up SIRI	2.24 ± 1.21	1.68 ± 1.21	0.0429 *
Preop SII	1736.78 ± 1730.19	1119.30 ± 1478.72	0.2682
Postop SII	3223.74 ± 3410.12	2735.26 ± 1192.49	0.4363
Follow-up SII	896.85 ± 364.45	758.91 ± 337.11	0.1548
Preop NLR	2.63 ± 0.66	2.54 ± 0.91	0.6829
Postop NLR	2.79 ± 0.83	2.34 ± 0.80	0.0476
Follow-up NLR	2.43 ± 0.91	2.32 ± 0.92	0.6404
Preop PLR	207.48 ± 53.54	201.61 ± 60.82	0.7819
Postop PLR	207.16 ± 57.49	197.33 ± 51.73	0.5121
Follow-up PLR	205.81 ± 62.80	192.50 ± 50.79	0.4889
Preop MLR	0.37 ± 0.08	0.34 ± 0.08	0.1999
Postop MLR	0.36 ± 0.09	0.34 ± 0.08	0.4902
Follow-up MLR	0.34 ± 0.07	0.35 ± 0.09	0.7688

Mann–Whitney U test (* *p* < 0.05).

## Data Availability

The data used in this study can be requested from the corresponding author.

## Abbreviations

LSS	Lumbar Spinal Stenosis
OVF	Osteoporotic Vertebral Fracture
SIRI	Systemic İnflammatory Response İndex
SII	Systemic İmmune-İnflammation İndex
NLR	Neutrophil-To-Lymphocyte Ratio
PLR	Platelet-To-Lymphocyte Ratio
MLR	Monocyte-To-Lymphocyte Ratio
